# Sonographic visualization of nipple blood flow can help differentiate Paget disease from benign eczematous nipple lesions

**DOI:** 10.1371/journal.pone.0197156

**Published:** 2018-05-16

**Authors:** Hideaki Ogata, Yukio Mitsuzuka, Naoko Honma, Miho Yoshida, Makoto Sumazaki, Fumi Saito, Masahiro Kobayashi, Kazutoshi Shibuya, Tetsuo Mikami, Hironori Kaneko

**Affiliations:** 1 Division of Breast and Endocrine Surgery (Omori), Department of Surgery, School of Medicine, Toho University, Omorinishi, Ota-ku, Tokyo, Japan; 2 Department of Clinical Physiology, Toho University Medical Center Omori Hospital, Omorinishi, Ota-ku, Tokyo, Japan; 3 Department of Pathology, School of Medicine, Toho University, Omorinishi, Ota-ku, Tokyo, Japan; 4 Department of Radiology, School of Medicine, Toho University, Omorinishi, Ota-ku, Tokyo, Japan; 5 Department of Surgical Pathology, School of Medicine, Toho University, Omorinishi, Ota-ku, Tokyo, Japan; University of South Alabama Mitchell Cancer Institute, UNITED STATES

## Abstract

**Purpose:**

Paget disease of the breast is a rare cancer that originates from the nipple–areolar complex. It is often overlooked and misdiagnosed as benign chronic eczema of the nipple. We aimed to retrospectively verify whether blood flow analysis using Doppler sonography was useful for detecting the presence of Paget disease.

**Methods:**

In this retrospective study, 12 patients with pathologically proven unilateral nipple eczematous lesions (seven with Paget disease and five with simple dermatitis) were included. Nipple blood flow signal was observed using Doppler sonography, and the detected blood flow signals were quantified using digitally recorded images. Quantified blood flow ratio and pathologically examined capillary density were evaluated between affected and unaffected nipples. Findings of mammography, grayscale sonography, and contrast-enhanced magnetic resonance imaging (CE-MRI) were reviewed.

**Results:**

In patients with Paget disease, Doppler effects in the affected nipple were more clearly visible than those in the unaffected nipple. These effects were sufficiently visible to identify Paget disease. No obvious effects were observed in the affected and unaffected nipples of simple dermatitis. The quantified blood flow ratio and pathologically examined capillary density were significantly higher for the Paget lesion than those for the non-Paget lesion. The sensitivity of CE-MRI and Doppler sonography was markedly correlated, revealing blood flow changes in the nipple lesions of Paget disease.

**Conclusion:**

Doppler sonography visualized the proliferation of blood vessels in Paget lesions. The visualization of increased nipple blood flow using Doppler sonography is a simple and low-cost method that provides useful data for identifying Paget disease during routine medical care.

## Introduction

Paget disease of the breast is a rare disorder of the nipple–areolar complex, accounting for 0.5%–5% of all breast carcinomas, and is characterized by an ulcerated, crusted, or scaling lesion on the nipple that extends to the areolar region [1, 2]. The prevalence of breast cancer associated with Paget disease is reportedly >90% [3, 4]. On the basis of its macroscopic features, Paget disease is often mistaken for benign skin conditions such as dermatitis [5–7], which may delay the accurate diagnosis of such lesions until they have progressed to an advanced stage and are more difficult to treat. Because mammography and grayscale sonography are not specific for the detection of Paget disease, they have limited value for diagnosing Paget disease. Contrast-enhanced magnetic resonance imaging (CE-MRI) is used for detecting underlying nipple lesions but is performed less frequently because of its cost and accessibility in an outpatient setting. Therefore, it is necessary to develop new diagnostic tools and methodologies to prevent misdiagnosis at the initial evaluation during routine medical care.

In this study, we verified whether nipple blood flow analysis using Doppler sonography was useful for detecting the presence of Paget disease.

## Patients and methods

The institutional review board of Toho University approved this retrospective study (approval number: M-16091), and patients’ informed consent was waived because the data were anonymously analyzed. The medical records of 12 patients who visited our facility from 2009 to 2014 for the treatment of a unilateral nipple–areolar lesion were retrospectively reviewed; seven patients with Paget disease (age range, 62–83 years; mean age, 76 years) and five with unilateral nipple dermatitis (age range, 35–74 years; mean age, 61 years) were included. Biopsy of the nipple–areolar lesion was performed after completing all imaging studies, and the diagnosis of all patients was pathologically confirmed. Patients with concurrent symptoms of infection were excluded.

Sonography was performed using the Aplio Diagnostic Ultrasound System with a 7–12-MHz linear array transducer (Toshiba Medical Systems, Otawara, Tochigi, Japan). All patients underwent color or power Doppler sonography to assess nipple vascularity. Blood flow of both nipples was estimated using Doppler sonography with standard instrument settings (flow speed, 3.8–5.8 cm/s; gain, 40–45). Images of the areas of maximum blood flow were obtained and digitally recorded to quantify nipple blood flow volume. Two sonologists who specialized in breast sonography evaluated the imaging findings by consensus. Mammograms were obtained using MMERX (Toshiba Medical Systems, Tsukuda, Tokyo, Japan). MRI was acquired using the GE Signa HDxt 1.5-T scanner (GE Medical Systems, Milwaukee, WI). A dedicated 8-channel breast coil was used with the patient in the prone position, and the following sequences were obtained: a post-contrast sagittal T1-weighted three-dimensional, fat-suppressed, fast gradient spoiled sequence (TR/TE, 4.6/2.2; flip angle, 10°; section thickness, 2.4 mm; acquisition time, 1 min) was obtained before and 30, 90, 150, 210, and 270 s after a rapid bolus injection of 0.2 mmol/kg body weight of Gd-DTPA (Magnevist, Schering, Berlin, Germany or Omniscan, Daiichisankyo, Tokyo, Japan).

### Quantification of nipple blood flow volume using Doppler sonography

**Fig 1:**
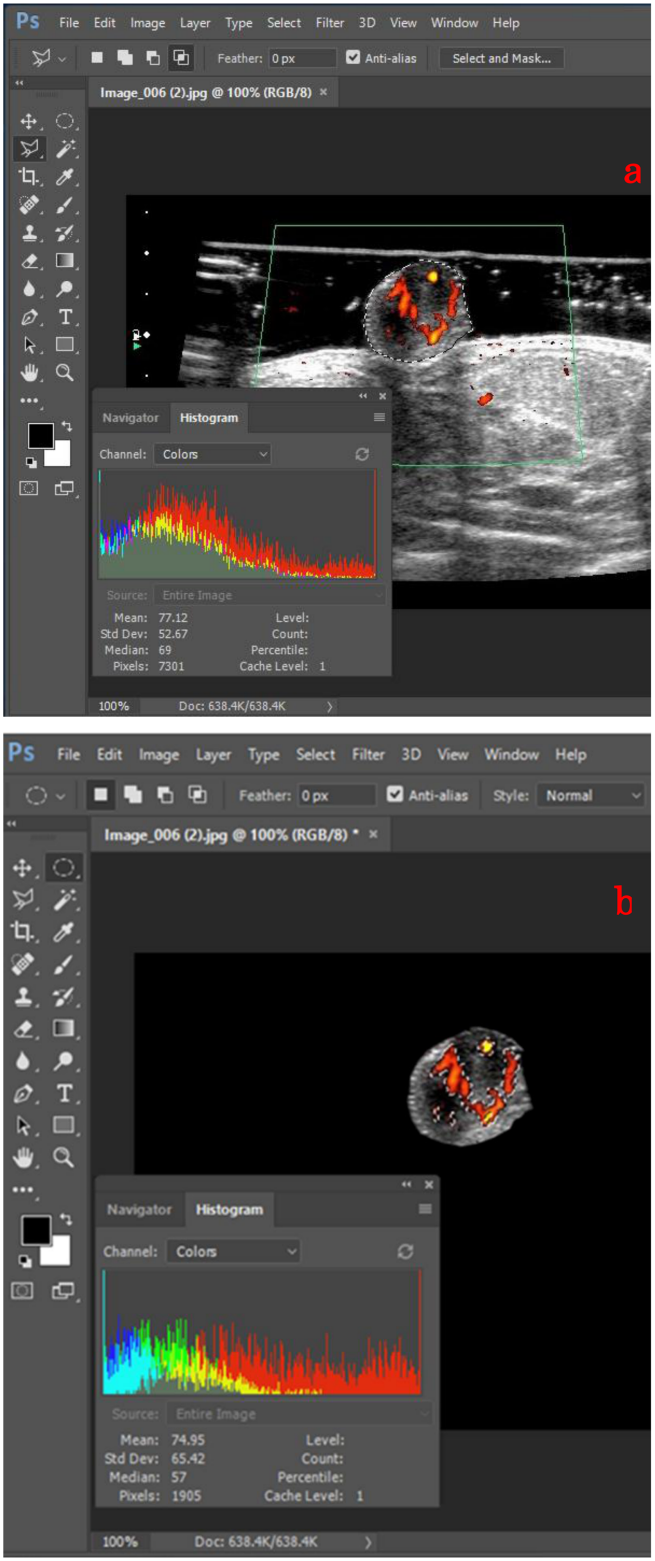
The area selection and calculation using Photoshop. (a) Using the “Magnetic Lasso” Tool, the nipple area was selected and calculated by counting the number of pixels enclosed in the area. (b) Using the “Similar” function, the whole blood flow signals within the traced nipple was defined and calculated by counting the number of pixels.

Using the Adobe Photoshop software (Adobe, San Jose, CA), the total number of pixels within the nipple and those indicating blood flow signals on the recorded Doppler sonograms were counted. First, the “Magnetic Lasso” Tool in Photoshop was used to delineate the boundary of the nipple area. The total number of pixels within the nipple was then counted using the “Histogram” function (Fig 1a). To define blood flow signals within the nipple, the nipple area was traced. After selecting a part of the blood flow signals in the traced nipple area using a selection tool, the whole blood flow signals within the nipple was defined with an approximate color using the “Similar” function. The number of pixels that indicated the blood flow was also counted using the “Histogram” function (Fig 1b). The nipple blood flow ratio was then calculated as the number of pixels that indicated the blood flow in the nipple divided by the total number of pixels in the nipple.

### Pathological examination

Surgically resected specimens of all seven cases of Paget disease and skin biopsies of three of the five nipple dermatitis cases were collected from the Pathology Department at our hospital. Two cases of dermatitis were excluded because of an insufficient quantity of specimen tissue for observing blood vessels within the dermis. Hematoxylin- and eosin-stained specimens were examined using a light microscope. The numbers of all capillaries and capillaries of >50-μm diameter within a 10-mm^2^ area of the dermis beneath the lesion (Paget disease or dermatitis) were counted using an ocular micrometer, and the number of capillary per mm^2^ was considered as the capillary density. In the case of Paget disease, normal skin at least 1 cm apart from the Paget lesion was examined as the control.

### Statistical analysis

All statistical analyses were performed using the JMP software version 10 (SAS Institute Inc., Cary, NC, USA). Quantitative data are presented as means ± standard deviations (SDs). A *p* value of <0.05 was considered to be statistically significant for all analyses. The Wilcoxon signed-rank test was used for blood flow analysis. Analysis of variance (ANOVA) was used to identify histological differences in capillary density, and Turkey–Kramer test was used for comparisons between the two groups.

## Results

The clinical and pathological findings of the 12 patients are shown in Table 1.

**Table 1:** Clinical and pathological findings of the 12 patients.

Case	Age (years)	Site	Symptoms of the nipple	Pathology
1	75	Lt	Nipple erosion	Paget disease with DCIS
2	78	Rt	Nipple itching and discoloration	Paget disease with DCIS
3	62	Rt	Nipple and areolar eczematous change and itching	Paget disease with DCIS
4	83	Rt	Nipple and areolar erosion	Paget disease
5	73	Lt	Nipple crusted change	Paget disease with IDC
6	83	Lt	Nipple and areolar eczematous change	Paget disease with IDC
7	81	Rt	Nipple erosion and scaling	Paget disease
8	69	Lt	Nipple eczematous change	Simple dermatitis
9	35	Lt	Nipple itching and discoloration	Simple dermatitis
10	74	Lt	Nipple itching and pigmentation	Simple dermatitis
11	68	Rt	Nipple erosion and bleeding	Simple dermatitis
12	59	Rt	Nipple itching	Simple dermatitis

The clinical presentation of the nipple–areolar region was variable, including erosion, itching sensation, and discoloration. Among the seven patients with Paget disease, three had ductal carcinoma in situ and two had invasive ductal carcinoma. In two patients, no evidence of underlying malignancy was found.

**Fig 2:**
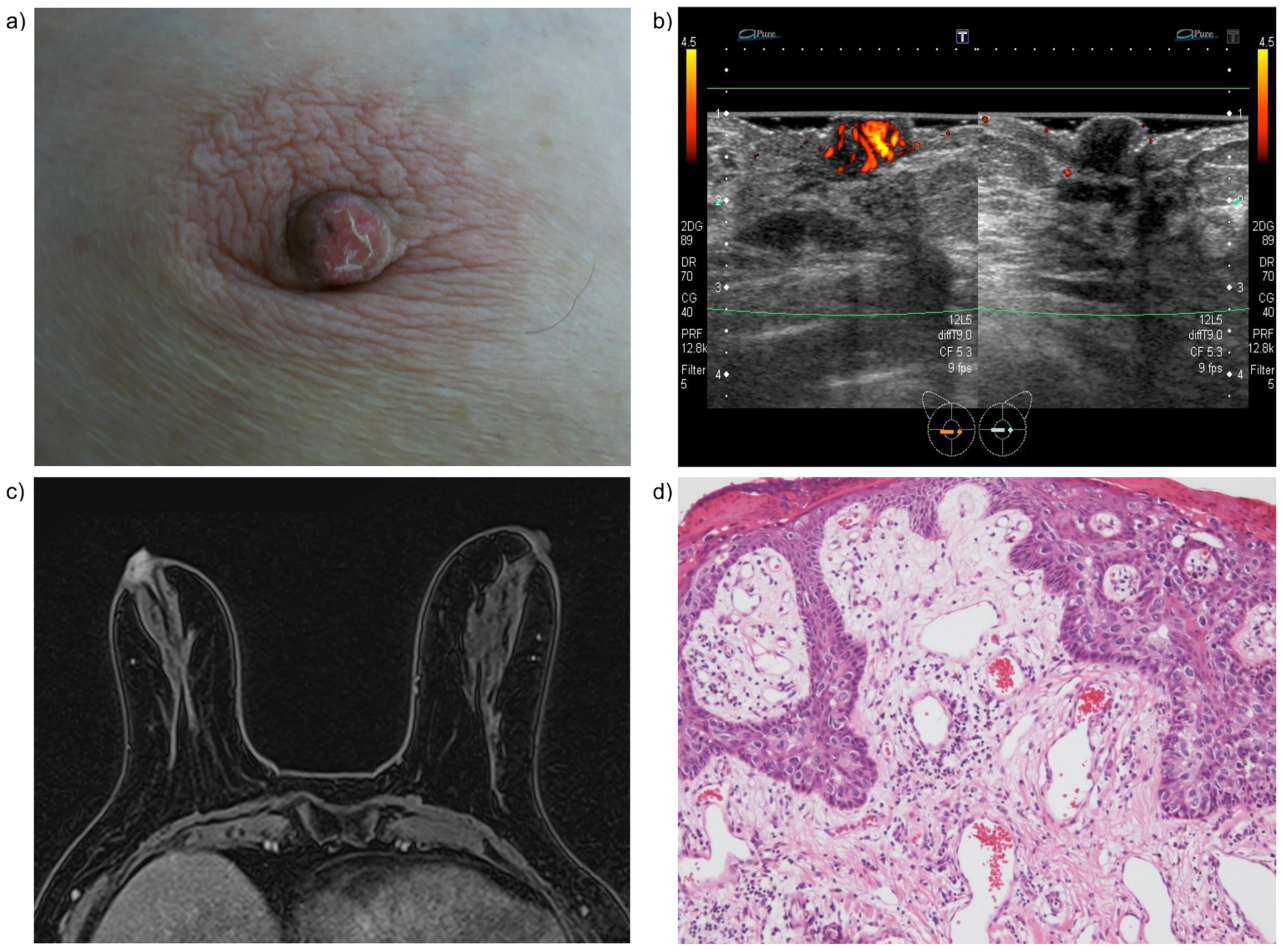
An 81-year-old woman with Paget disease (Case 7). (a) Scaling and erosion of the affected right nipple. (b) Blood flow signals inside the affected nipple as observed using Doppler sonography. (c) Nipple enhancement by CE-MRI. (d) Histopathological examinations with hematoxylin and eosin staining reveal Paget cells within the epidermis and capillary proliferation within the dermis (×100).

Fig 2 shows a case of an 81-year-old woman with Paget disease (Case 7). Because neither mammography nor grayscale sonography revealed any findings, obvious blood flow signals inside the affected nipple, detected using Doppler sonography, was the only image that suggested latent disease during the initial evaluation of ambulatory practice. On the basis of the Doppler sonography results, was performed, and Paget disease was pathologically diagnosed.

**Fig 3:**
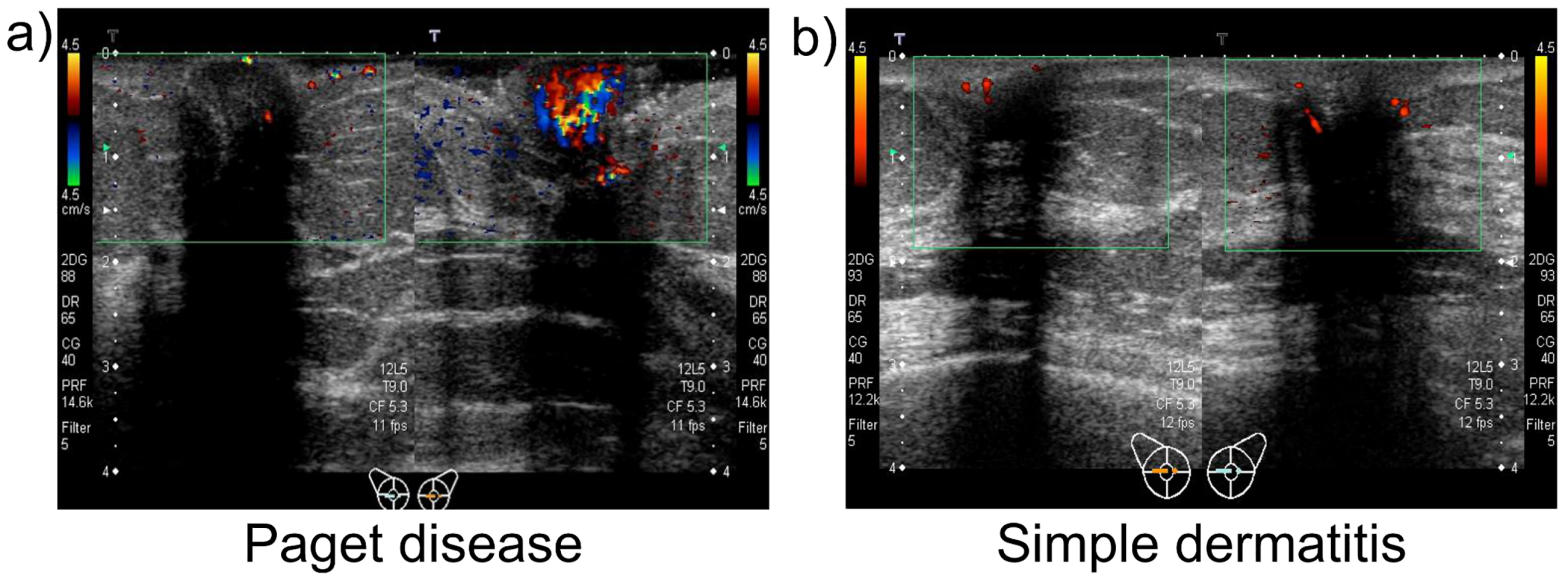
(a) Visualized hypervascularity of the affected nipple with Paget disease (Case 1). (b) Unchanging blood flow signals of the affected nipple with simple dermatitis (Case 8).

Fig 3 indicates the difference of Doppler effects between Paget disease and benign simple dermatitis. Typical images of Doppler sonograms of a patient with Paget lesions of the left nipple (Case 1) are shown in Fig 3a. Increased blood flow signaling within the affected nipple was observed using Doppler sonography. Here, the difference in blood flow signals between the affected and unaffected nipples was strikingly obvious. The visualization of these Doppler effects was highly reproducible and was observed in the affected nipple in all seven cases of Paget disease. Fig 3b shows images of a patient with simple dermatitis of the left nipple (Case 8). No apparent blood flow signals were visualized in the affected and unaffected nipples using Doppler sonography. Furthermore, no apparent blood flow signals were detected in the affected nipple of the other four cases with simple dermatitis.

**Fig 4:**
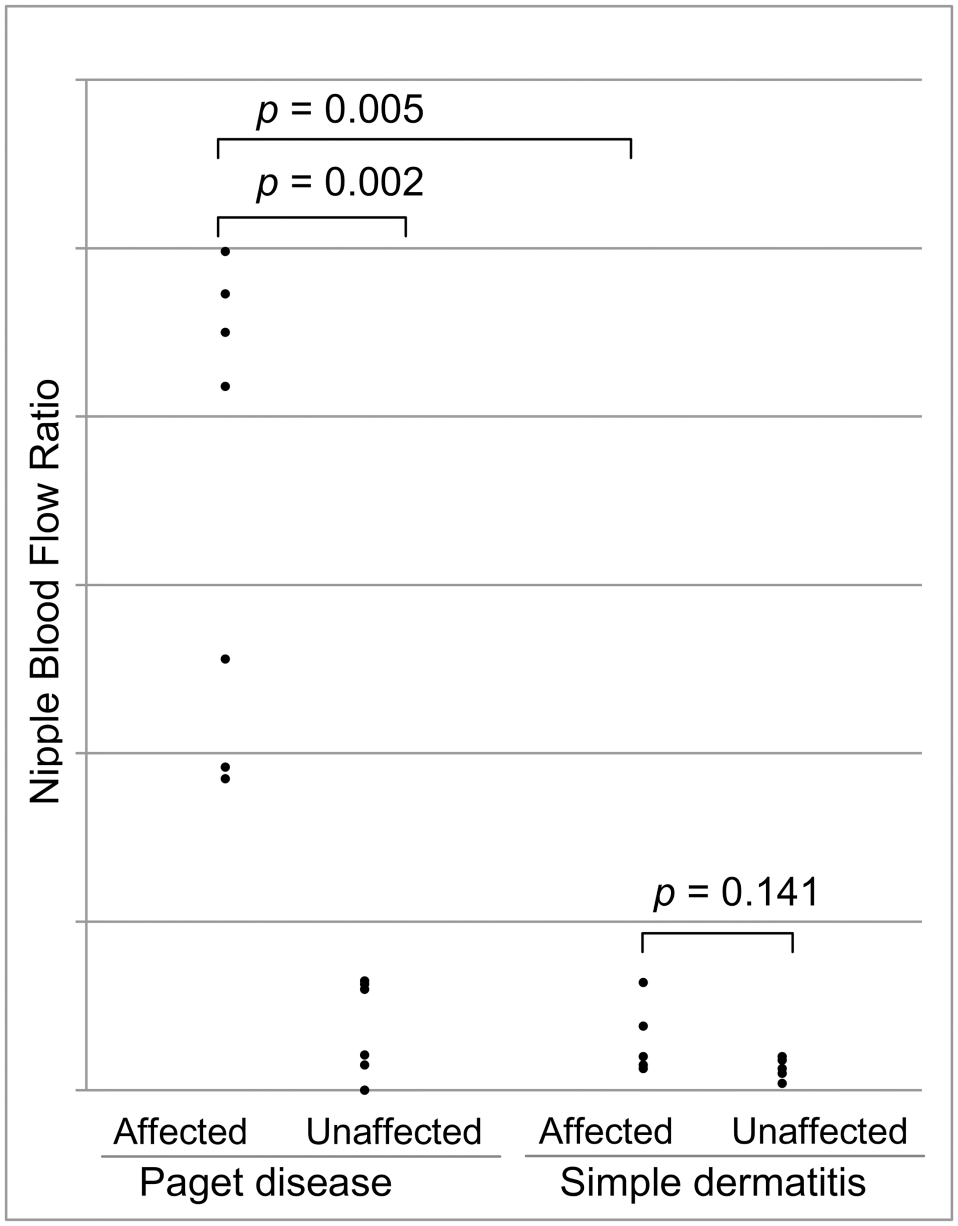
Comparison of nipple blood flow ratios between the affected and unaffected nipples with each condition.

Fig 4 shows the comparison of nipple blood flow ratios (blood flow pixel count/total pixel count) between the affected and unaffected nipples with each condition. In Paget disease, the mean (±SD) nipple blood flow ratio was significantly greater in the affected nipple than in the unaffected nipple (mean ± SD, 0.353 ± 0.213 vs. 0.032 ± 0.0297; *p* = 0.002). In simple dermatitis, no significant difference was observed between the affected and the unaffected nipples (0.03 ± 0.021 vs. 0.013 ± 0.0064; *p* = 0.141). In addition, when blood flow ratios of the affected nipple with Paget disease and simple dermatitis were compared, the mean (±SD) nipple blood flow ratio of the affected nipple with Paget disease was significantly greater than that of the affected nipples with simple dermatitis (0.353 ± 0.213 vs. 0.030 ± 0.021, *p* = 0.005) (S1 Table).

**Fig 5:**
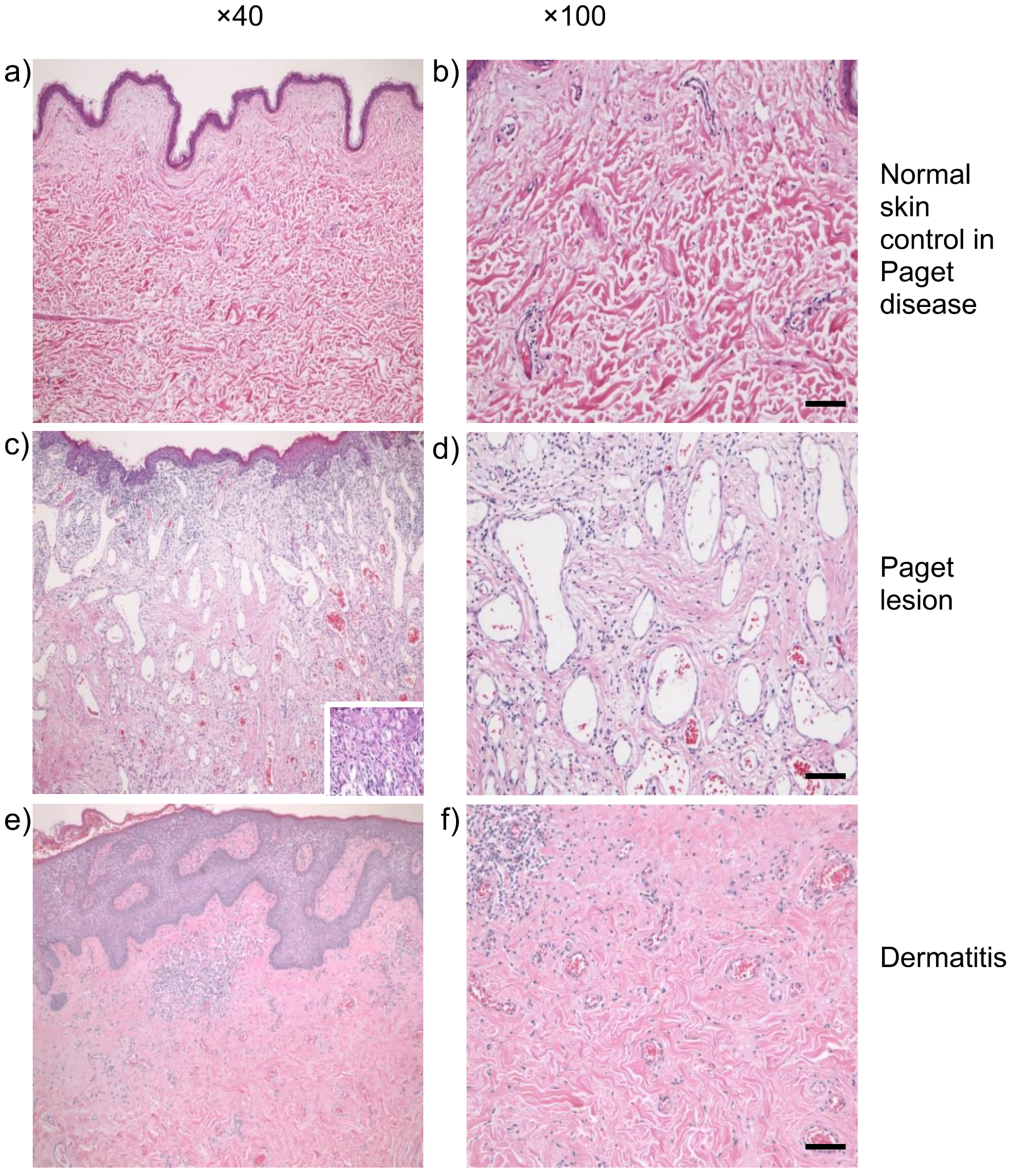
Histopathological examinations of capillary proliferation of the nipple–areolar region by hematoxylin and eosin staining. (a) Normal skin control with Paget disease (×40). (b) Normal skin control with Paget disease (×100). (c) Paget lesion at ×40). (d) Paget lesion at ×100). (e) Dermatitis at ×40). (f) Dermatitis at ×100). Bar, 50 μm (b, d, and f).

**Fig 6:**
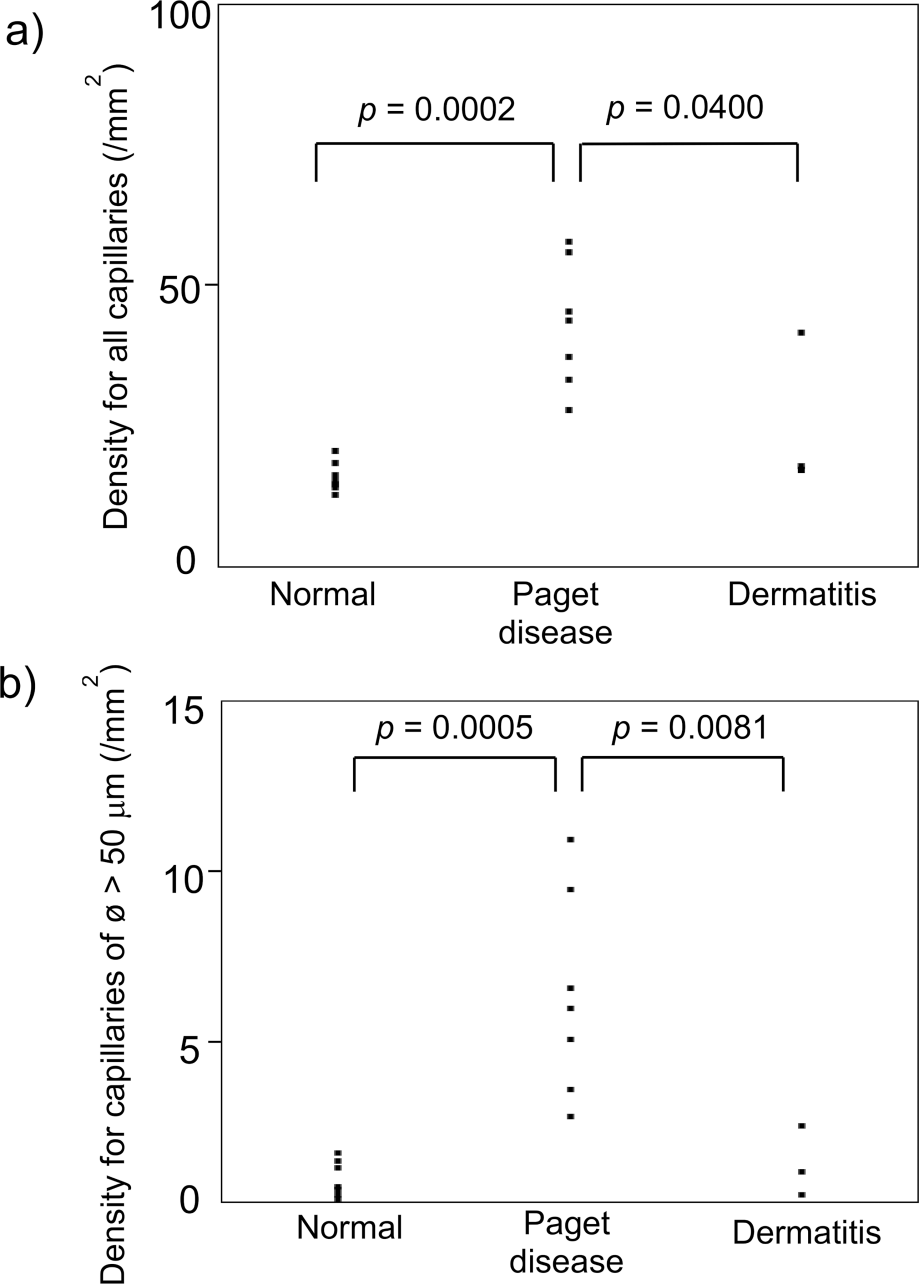
Comparison of the capillary density (a, all; b, ø > 50 μm) by histological examination. ANOVA, Paget disease vs. dermatitis: *p* = 0.0003 for all capillaries (a) and *p* = 0.0004 for capillaries thicker than 50 μm (b). The mean (±SD).

Pathological examination results revealed that capillaries increased in both number and diameter within the dermis beneath the Paget lesion (Fig 5). The capillary density (mean ± SD/mm^2^) was significantly different according to the histology, irrespective of the capillary thickness (ANOVA; *p* = 0.0003 for all capillaries and *p* = 0.0004 for capillaries thicker than 50 μm). The mean (±SD) capillary density of the Paget lesions was significantly higher than that of normal skin control specimens and dermatitis (43.0 ± 11.2 vs. 15.9 ± 2.7 and 25.7 ± 13.7; *p* = 0.0002 and 0.0400, respectively; Fig 6a) (S2 Table). Moreover, when limited to capillaries of >50 μm in diameter, the mean (±SD) capillary density of Paget lesions was significantly higher than that of normal skin control specimens and dermatitis (6.2 ± 3.0 vs. 0.7 ± 0.5 and 1.1 ± 1.0; *p* = 0.0005 and 0.0081, respectively; Fig 6b) (S3 Table).

The findings of mammography, grayscale sonography, and CE-MRI for seven patients with Paget disease were reviewed (Table 2). Mammography and grayscale sonography were performed for all seven patients. CE-MRI images were obtained for six patients. Mammograms revealed the types of microcalcifications in four patients, parenchymal mass or asymmetry in two, and areolar thickening that suggested Paget disease in one. Grayscale sonograms showed the types of microcalcifications in four patients, parenchymal mass or asymmetry in two, and nipple or areola thickening that suggested Paget disease in three. CE-MRIs showed clear enhancement of the affected nipples in all six patients.

**Table 2:** The findings of mammography, grayscale sonography, and CE-MRI for seven patients with Paget disease.

	positive
Mammogram Microcalcifications Nipple morphologic change Asymmetry or mass	4/7 (57%) 1/7 (14%) 2/7 (28%)
Greyscale-sonogram Microcalcifications Nipple morphologic change Asymmetry or mass	4/7 (57%) 3/7 (42%) 2/7 (28%)
Nipple enhancement on CE-MRI	6/6 (100%)
Visualized blood flow on Doppler sonogram	7/7 (100%)

## Discussion

To establish a diagnosis of Paget disease, a full-thickness biopsy of the nipple and areolar region is essential [8]. However, the criteria to accurately diagnose suspected Paget disease in patients with nipple and areolar changes remain unclear. The observation of disease progression with or without the topical application of corticosteroids is often helpful for distinguishing a malignant lesion. However, in Paget disease, a delayed diagnosis that results from treatment with topical steroids aggravates the underlying malignancy [5]. Therefore, specific criteria to differentiate Paget disease from benign nipple lesions are required for accurate initial evaluations during routine medical care.

Although morphological inspection (including visual inspection, mammography, and grayscale sonography) offers no specific findings for differentiating Paget disease from benign nipple lesions [9–12], the higher sensitivity of CE-MRI can provide critical data for confirming Paget disease [13], with 100% enhancement of nipples with Paget disease being reported using CE-MRI [9, 14, 15]. In this study, grayscale sonography detected morphological changes of the nipple, suggestive of Paget disease, in three of seven patients, and mammography detected the same in one patient only; however, enhancement of the affected nipple was observed in all six patients assessed using CE-MRI. Moreover, nipple blood flow in these patients was assessed using Doppler sonography. These findings indicated that CE-MRI and Doppler sonography were both comparably sensitive for detecting blood flow characteristics of nipples with Paget lesion.

Although the use of CE-MRI is justified for detecting Paget lesions, its use for breast screening of all patients with nipple–areolar lesions (including benign lesions) is not practical because of the high cost and low accessibility in ambulatory practice [16]. As an alternative, nipple blood flow visualization using Doppler sonography is easy to perform in an outpatient setting and shows highly reproducible results because of the small display area and limited focus on the body surface. Therefore, examining nipple blood flow using Doppler sonography should be performed first in routine medical care for patients with nipple and areolar changes, which are consistent with Paget disease.

In dermatology, Doppler sonograms have been evaluated in the past decades; the sensitivity and specificity of hypervascularity detected using Doppler sonography in malignant skin tumors were 90% and 100%, respectively, whereas those of hypovascularity in benign lesions were 100% and 90%, respectively [17]. Particularly in malignant melanoma, higher blood flow signals detected by Doppler sonography is useful for differentiation from benign lesions [18, 19]. Angiogenesis is indispensable for the proliferation of malignant tumors, including breast cancer [20], although no reports analyzed the abnormal capillary formation of Paget lesion. This report describes a new sonographic finding that can be observed in patients with Paget disease. It should be emphasized that this finding should not be used in isolation for diagnosis but should be used as a base for making a decision to perform nipple biopsy.

Because Paget disease of the breast is rare [1], our study cohort was relatively small. Nonetheless, this study is the first to systematically investigate the blood flow status of the nipple–areolar region of Paget disease using Doppler sonography. Considering these remarkable and encouraging results, open label clinical studies of the efficacy of Doppler sonography in this population are clearly warranted.

## Supporting information

S1 Table (DOCX)

S2 Table (DOCX)

S3 Table (DOCX)
