# Supplementary material for: Sonographic visualization of nipple blood flow can help differentiate Paget disease from benign eczematous nipple lesions
Source: PLoS One. 2018 May 16;13(5):e0197156. doi: 10.1371/journal.pone.0197156 (PMC5955580; doi:10.1371/journal.pone.0197156)
Supplement: S1 Table — (DOCX) [file pone.0197156.s001.docx]

**Supporting Information**

<This table is relevant to Fig 4>

**S1 Table. Nipple blood flow ratio**

| **Case#** | **Age (years)** |  | **Affected nipple blood flow ratio** | **Unaffected nipple blood flow ratio** | **Condition** |
| --- | --- | --- | --- | --- | --- |
| 1 | 75 |  | 0.473 | 0.015 | Paget disease |
| 2 | 78 |  | 0.192 | 0.021 | Paget disease |
| 3 | 62 |  | 0.256 | 0.065 | Paget disease |
| 4 | 83 |  | 0.498 | 0.06 | Paget disease |
| 5 | 73 |  | 0.185 | 0.063 | Paget disease |
| 6 | 83 |  | 0.45 | 0 | Paget disease |
| 7 | 81 |  | 0.418 | 0 | Paget disease |
| 8 | 69 |  | 0.015 | 0.004 | Dermatitis |
| 9 | 35 |  | 0.038 | 0.018 | Dermatitis |
| 10 | 74 |  | 0.013 | 0.013 | Dermatitis |
| 11 | 68 |  | 0.02 | 0.02 | Dermatitis |
| 12 | 59 |  | 0.064 | 0.01 | Dermatitis |
