# Supplementary material for: Sonographic visualization of nipple blood flow can help differentiate Paget disease from benign eczematous nipple lesions
Source: PLoS One. 2018 May 16;13(5):e0197156. doi: 10.1371/journal.pone.0197156 (PMC5955580; doi:10.1371/journal.pone.0197156)
Supplement: S2 Table — (DOCX) [file pone.0197156.s002.docx]

<This table is relevant to Fig 6a>

**S2 Table. Pathologically examined densities of capillaries (/mm^2^)**

| **Case#** | **Age (years)** | **Histological type** | **Vascular type** | **Density** | | **Mean**  **(/mm^2^)** |
| --- | --- | --- | --- | --- | --- | --- |
|  |  |  |  | **Pathologist 1(/mm^2^)** | **Pathologist 2(/mm^2^)** |  |
| 1 | 75 | Paget | All | 45 | 45.7 | 45.35 |
|  |  | Normal | All | 15.2 | 9.9 | 12.55 |
| 2 | 78 | Paget | All | 34.5 | 32 | 33.25 |
|  |  | Normal | All | 14.6 | 15.8 | 15.2 |
| 3 | 62 | Paget | All | 22.3 | 33.2 | 27.75 |
|  |  | Normal | All | 18.1 | 22.7 | 20.4 |
| 4 | 83 | Paget | All | 44.3 | 43.3 | 43.8 |
|  |  | Normal | All | 13.5 | 14.3 | 13.9 |
| 5 | 73 | Paget | All | 64.1 | 51.4 | 57.75 |
|  |  | Normal | All | 19.3 | 17.6 | 18.45 |
| 6 | 83 | Paget | All | 56.2 | 55.3 | 55.75 |
|  |  | Normal | All | 11.9 | 17.1 | 14.5 |
| 7 | 81 | Paget | All | 42.6 | 31.7 | 37.15 |
|  |  | Normal | All | 14.6 | 18 | 16.3 |
| 8 | 69 | X (Other disease) | All | 14.6 | 21 | 17.8 |
| 9 | 35 | X (Other disease) | All | 21.9 | 13.9 | 17.9 |
| 10 | 74 | X (Other disease) | All | 38.7 | 44.3 | 41.5 |
