# Supplementary material for: Sonographic visualization of nipple blood flow can help differentiate Paget disease from benign eczematous nipple lesions
Source: PLoS One. 2018 May 16;13(5):e0197156. doi: 10.1371/journal.pone.0197156 (PMC5955580; doi:10.1371/journal.pone.0197156)
Supplement: S3 Table — (DOCX) [file pone.0197156.s003.docx]

<This table is relevant to Fig 6b>

**S3 Table. Pathologically examined densities of capillaries with ø > 50 μm (/mm^2^)**

| **Case#** | **Age (years)** | **Histological type** | **Vascular type** | **Density** | | **Mean**  **(/mm^2^)** |
| --- | --- | --- | --- | --- | --- | --- |
|  |  |  |  | **Pathologist 1(/mm^2^)** | **Pathologist 2(/mm^2^)** |  |
| 1 | 75 | Paget | Thick | 5 | 6.6 | 5.8 |
|  |  | Normal | Thick | 0.2 | 0 | 0.1 |
| 2 | 78 | Paget | Thick | 2.2 | 2.9 | 2.55 |
|  |  | Normal | Thick | 0.1 | 0.6 | 0.35 |
| 3 | 62 | Paget | Thick | 3 | 3.7 | 3.35 |
|  |  | Normal | Thick | 1.1 | 0.9 | 1 |
| 4 | 83 | Paget | Thick | 5.1 | 7.7 | 6.4 |
|  |  | Normal | Thick | 0.3 | 0.1 | 0.2 |
| 5 | 73 | Paget | Thick | 7.9 | 10.8 | 9.35 |
|  |  | Normal | Thick | 0.5 | 0.4 | 0.45 |
| 6 | 83 | Paget | Thick | 12 | 9.7 | 10.85 |
|  |  | Normal | Thick | 0.7 | 2.2 | 1.45 |
| 7 | 81 | Paget | Thick | 3.4 | 6.3 | 4.85 |
|  |  | Normal | Thick | 0.8 | 1.6 | 1.2 |
| 8 | 69 | X (Other disease) | Thick | 0.1 | 0.3 | 0.2 |
| 9 | 35 | X (Other disease) | Thick | 1 | 0.8 | 0.9 |
| 10 | 74 | X (Other disease) | Thick | 1.3 | 3.2 | 2.25 |
